# A Randomized Controlled Trial on the Effects of Anticipated Regret on Pro-Environmental Behaviors

**DOI:** 10.3390/bs16050664

**Published:** 2026-04-28

**Authors:** Aurora Bonvino, Eugenio Trotta, Gianluigi Serio, Loreta Cannito, Tiziana Quarto, Paola Palladino

**Affiliations:** 1 Applied Experimental Psychology Lab, University of Foggia, 71121 Foggia, Italy; aurora.bonvino@uniba.it (A.B.); eugenio.trotta@unifg.it (E.T.); gianluigi.serio@unifg.it (G.S.); loreta.cannito@unifg.it (L.C.); paola.palladino@unifg.it (P.P.); 2 Department of Translational Biomedicine and Neuroscience, University of Bari, 70124 Bari, Italy

**Keywords:** anticipated regret, pro-environmental behavior, EkoTok, young adults

## Abstract

Environmental sustainability is crucial for human survival and the future of new generations. Anticipating regret can influence decision-making and promote sustainable behaviors. This study examines the effect of anticipated regret on pro-environmental behaviors among young adults (18–30) using regret-based short videos called “EkoToks.” A total of 128 participants were randomly assigned to an experimental group, receiving regret-evoking videos, or a control group, receiving informational videos. Pro-environmental behaviors were measured at baseline, post-test, and at three-month follow-up. Results showed significant short-term improvements in the experimental group compared to the control group, with higher scores in total pro-environmental behavior, prosocial behavior, reuse, recycling, and pro-environmental actions. At follow-up, the experimental group continued to outperform the control group in terms of total behavior, prosocial behavior, recycling, reuse, pro-environmental actions, and waste reduction. Regression analyses revealed that post-test regret significantly predicted further improvements at follow-up (compared to post-test) in total behavior, prosocial behavior, reuse, and pro-environmental actions. These findings highlight the effectiveness of anticipated regret in improving environmental behaviors, particularly low-cost ones.

## 1. Introduction

Environmental sustainability is crucial for human survival and future generations. Several studies highlight the link between environmental challenges and human behavior, encouraging individuals, groups, and communities to reduce their ecological footprint through pro-environmental actions (Appannagari, 2017; Cook et al., 2013; Trenberth, 2018). Despite the growing interest in sustainability, many people do not adopt “green habits” (O’Brien, 2008). Thus, psychological research is increasingly focused on understanding the factors that drive pro-environmental actions to develop effective interventions. These efforts align with the 17 Sustainable Development Goals (SDGs) outlined in the 2030 Agenda (see Steiner et al., 2018).

Pro-environmental behavior includes actions like recycling, minimizing energy consumption, reusing items, buying eco-friendly products, and participating in pro-environmental actions—all of which aim to reduce individuals’ impact on the environment. Recent studies suggest that pro-environmental behavior may emerge early in development (for example, see Hu & Wu, 2022; Geraci et al., 2024). Geraci et al. (2023) showed that 7-month-old infants already display a preference for agents engaging in explicit pro-environmental actions. Specifically, infants preferred agents who collected waste from the environment compared to those who ignored it, indicating a rudimentary form of environmental moral evaluation. This early sensitivity to environmental actions underscores the importance of considering both cognitive and socioemotional processes in the development of ecological responsibility. As suggested by Geraci et al. (2024), children progressively refine their capacity to evaluate different types of pro-environmental behaviors, distinguishing not only between harmful and beneficial actions, but also between the underlying intentions and their consequences. Further studies on environmental education highlighted that both environmental knowledge and connectedness to nature play a central role in fostering ecological behavior during development (Otto & Pensini, 2017). Longitudinal studies showed that childhood experiences with nature and family environmental values can predict environmentally responsible behavior in adulthood (Evans et al., 2018).

Kollmuss and Agyeman (2002) provided a comprehensive model to explain the factors influencing pro-environmental behaviors. This model incorporates various theories and integrates external factors, such as socio-demographic, institutional, economic, social, and cultural aspects, as well as internal factors, including motivation, knowledge, awareness, values, and emotions. Kollmuss and Agyeman’s model effectively clarifies the disparity between acquiring environmental knowledge and demonstrating pro-environmental actions. For instance, according to Kempton et al. (1995), environmental awareness alone is not sufficient for pro-environmental behavior due to cognitive and emotional limitations. Regarding cognitive limitations, the non-immediacy of the many environmental issues included makes them intangible and difficult to grasp emotionally. Furthermore, it is also considered the gradual nature of ecological destruction, which humans are not good at perceiving, and the complexity of environmental systems, leading people to adopt simpler mental shortcuts. Emotionally, people may not feel invested due to three conditions: (i) a lack of awareness of the causes and consequences of environmental degradation, (ii) resistance to information that conflicts with personal values, and (iii) the adoption of immature defense mechanisms to cope with painful feelings related to environmental issues. These defense mechanisms—such as apathy, denial, and detachment—often hinder individuals from adopting environmentally friendly behaviors. On the other hand, counterfactual emotions, such as regret, have been found to be more effective than negative emotions like fear or anger in motivating pro-environmental behavior (Chawla, 1999; Newhouse, 1990).

Regret has been central to decision-making theories, starting with Savage’s “minimax regret rule”, which suggests that individuals aim to minimize potential regret when making choices (Savage, 1972). Building on this foundation, Loomes and Sugden’s “regret theory” further illustrated the psychological experience of regret as a driving force in decision-making (Loomes & Sugden, 1982). Expanding these ideas, Zeelenberg and Pieters (2007) developed their “regret regulation theory,” explaining that regret is a cognitive emotion that helps individuals learn from past decisions to optimize future outcomes. Unlike other emotions, regret involves self-reflection on missed opportunities, promoting behavior changes (Lecci et al., 1994). Prior research has shown that emphasizing anticipated regret can influence behaviors in areas such as alcohol consumption, drug use, junk food intake, speeding, and safe sex practices, encouraging more responsible decision-making (Parker et al., 1996; Richard et al., 1996). Onwezen et al. (2014) compared the predictive power of different moral emotions, including pride, guilt, and anticipated regret, in driving pro-environmental behaviors. While both pride and guilt influenced sustainable actions, anticipated regret was found to be the strongest predictor. Carfora et al. (2017) reported the effect of anticipated regret on behaviors like increasing fruit and vegetable consumption, promoting water intake (Carfora et al., 2018), and reducing red meat consumption (Carfora et al., 2019) among young adults. Their studies used a combination of regret manipulation and informational messages to test the effects on participants’ behavior. For instance, in the red meat study, participants in the emotional condition received regret-inducing messages about the consequences of meat consumption, while those in the control group received neutral messages. Results showed that participants exposed to emotional messages had a significant decrease in red meat consumption compared to those exposed to purely informational messages.

Carfora et al. (2017) examined the impact of anticipated regret on consumption behavior, which represents only one aspect of pro-environmental behavior. While the study successfully demonstrated that anticipated regret could change specific behaviors, it did not comprehensively cover other domains of sustainable behavior, such as mobility or waste reduction. Therefore, additional research is needed to assess whether regret can be an effective motivator across a wider range of pro-environmental behaviors.

Although recent studies (Hysa et al., 2021; Schmidt et al., 2022) suggest that visual and interactive content is more impactful than traditional messaging for encouraging sustainable behaviors, the integration of mobile-based video interventions with the experimental manipulation of emotions, in the context of sustainability research, remains underexplored. This study advances this approach by using EkoToks, short, engaging videos combined with regret-based emotional triggers, which offer a more effective strategy for behavior change compared to traditional, text-based interventions like those used by Carfora et al. (2019). While Carfora and colleagues sent daily chatbot messages to influence behaviors such as reducing meat consumption, the EkoTok videos in this study provide a richer, more immersive experience, enhancing emotional engagement and making environmental consequences feel more immediate. The term refers to the communicative format of the intervention, which is inspired by the style of short-form social media videos (e.g., TikTok), characterized by brief duration, visual immediacy, and high engagement. This novel method is particularly well-suited to younger generations, who are more responsive to interactive and visual stimuli compared to passive messaging. Young adults, who possess higher neuroplasticity, are especially adaptable to interventions involving technology. Furthermore, in the context of sustainability, younger people—as opposed to children—go through a phase of separation from caregivers and learn how to live independently and autonomously, including decision-making and managing their daily environmental impact (Bradshaw et al., 2013; Spear, 2013).

### Aims

Although previous research has highlighted the importance of emotions in influencing sustainable decision-making, the role of anticipated regret in environmental communication remains relatively underexplored. This study aims to investigate the role of anticipated regret as a motivational mechanism for pro-environmental behaviors among young adults. Since limited attention has been given to how emotionally framed messages can influence everyday environmental behaviors, we introduced “EkoTok”—short, engaging videos delivered through automated messaging—to overcome psychological barriers (i.e., the intangibility of environmental consequences) and promote pro-environmental actions. The key strength of this study lies in integrating emotional theories with modern communication technologies focused on a specific population (young adults) uniquely positioned to make impactful decisions that can shape a more sustainable future through individual choice.

## 2. Methods

### 2.1. Research Design

The study protocol was approved by the Local Ethics Committee (protocol code 016/CEpsi—29 June 2023). Undergraduate students at the University of Foggia participated as volunteers at the study.

#### 2.1.1. Screening and Exclusion Criteria

Only participants aged between 18 and 30 years were included. Screening was conducted excluding prior psychopathological diagnoses, current drug treatment, current acute psychopathological symptoms, and/or special dietary regimens (diets, intolerances, vegan diet, vegetarian diet). Additional exclusion criteria were the presence of high levels of anxiety and/or prodromal signs of eating disorders, as measured respectively by the Self-Rating Anxiety Scale (SAS; Innamorati et al., 2006; Zung, 1971) and the Eating Attitudes Test-26 (EAT-26; Dotti & Lazzari, 1998; Garner et al., 1983). The Anxiety index was calculated using the Self-Rating Anxiety Scale and only participants with a score lower than 45 were included in the study (Zung, 1971). This choice was primarily driven by ethical considerations, as the planned experimental manipulation involved exposure to emotional content potentially harmful to the psychophysical well-being of participants experiencing acute phases of subclinical and/or clinical anxiety syndromes. The choice to exclude participants with scores above 20 on the EAT-26, as well as vegans or vegetarians, was made to minimize possible sample-related biases in pro-environmental measures related to food consumption.

#### 2.1.2. Procedure

**Figure 1:**
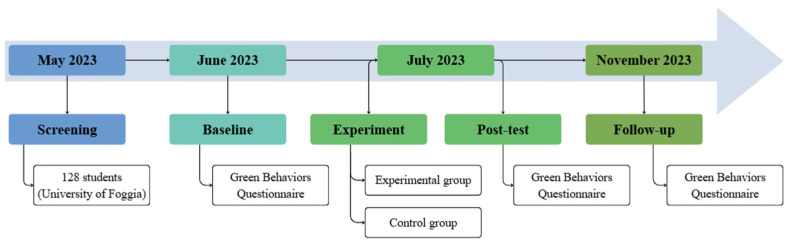
Longitudinal study design with initial screening, baseline, experimental phase, and follow-up assessments of pro-environmental behavior in both the EG and the CG.

At baseline (see Section 2.3 “Materials”), participants were randomly divided into the experimental group (EG) and the control group (CG). Preliminary analyses confirmed that the two groups did not significantly differ in age, gender, or socio-economic status (SES). During the experimental phase, all participants received, for ten consecutive days, an EkoTok (a short video delivered via an automated mobile chat; see Section 2.3.2) in the morning (9 a.m.). While the EG received the same educational videos with anticipated regret manipulation, the CG received the videos without any experimental manipulation. At the end of each video, participants in both groups were instructed to answer a control question. Pro-environmental behavior was assessed using an ad hoc questionnaire in both groups at post-test and at a three-month follow-up. Figure 1 shows the longitudinal design of the study.

### 2.2. Sample

A priori power analysis was conducted using G*Power version 3.1, assuming a statistical power of 0.95, an alpha level of 0.05, and a target effect size of 0.25 for repeated measures design with three within-subject time points and two between-subject groups. The analysis indicated a required minimum sample size of 124 participants. A total of 128 participants were recruited, and socio-demographic data were collected (Table 1). The sample was predominantly female (71%), with a mean age of approximately 23 years. Most participants lived with their parents (78%) and 61% reported not being responsible for daily shopping activities. Parental SES was assessed using the Hollingshead four-factor index (Hollingshead, 1975). It provides a score based on the parental level of education, occupation, gender, and marital status. For each participant, we considered the number of inhabitants associated with their residence, distinguishing those who live in an urban context (>15,000 inhabitants) and those in a rural context (<15,000 inhabitants). Most participants resided in urban areas (72%).

### 2.3. Materials

#### 2.3.1. Self-Report Measures

The Green Behaviors Questionnaire assesses different subscales associated with sustainable behavior (prosocial behavior, purchase, consume, reuse, recycling, waste reduction, mobility, and pro-environmental actions) and was developed ad hoc based on a review of the literature on the topic (De Young, 1985; Kaiser et al., 1999; Lange & Dewitte, 2019; Markle, 2013; Tilikidou et al., 2002). The subscales were defined based on the main behavior targeted by each item within the consumption–waste cycle. Although some conceptual overlap between domains (e.g., recycling, reuse, and waste reduction) is possible, each item was assigned to the dimension that best reflects its dominant behavioral function. The questionnaire consisted of 64 items (Table 2).

Each item response was rated on a Likert scale ranging from 1 (Never) to 5 (Always). The prosocial behavior score was derived from the sum of items 1–8; the purchase score was derived from the sum of items 9–16; the consume score was derived from the sum of items 17–24; the reuse score was derived from the sum of items 25–32; the recycling score was derived from the sum of items 33–40; the waste score was derived from the sum of items 41–48; the mobility score was derived from the sum of items 49–56; and the pro-environmental actions score was derived from the sum of items 57–64. Higher scores correspond to more favorable behaviors for the specific scale. The overall score was computed as the sum of the items, with higher scores indicating more pro-environmental behaviors. The scale showed an average score of 155 with a standard deviation (SD) of 19.38, indicating some variability in responses. The reliability of the scale was confirmed by Cronbach’s alpha (α) of 0.812, which suggested good internal consistency. Additionally, McDonald’s omega (ω) was slightly higher at 0.827, further supporting the scale’s reliability in measuring the construct.

The Marlowe–Crowne Social Desirability Scale (MC-SDS) was administered to quantify social desirability through self-assessment. The score obtained on this measure was used as a covariate in the statistical analyses.

#### 2.3.2. EkoTok

For 10 consecutive days, participants were invited to watch EkoToks (average duration 90 s) addressing specific pro-environmental behaviors (See Table A1 in Appendix A for description of the EkoTok messages). Each EkoTok was designed to highlight the human impact on the environment and the immediate and future environmental consequences of specific behaviors. In the EkoTok delivered to the EG, these consequences were introduced following the statement “*You may regret…*”. Participants in the CG viewed short informational videos on the same topics, without any reference to anticipated regret. The visual materials and informational content were the same; the only difference concerned the additional segment introducing the regret manipulation. Specifically, in the EG videos, the message “*You may regret…*” was displayed on a black screen before presenting the consequences of the behavior. After viewing all EkoToks, two ad hoc scales were administered, each consisting of 14 items, to evaluate post-experiment psychological outcomes: a scale on the perceived regret (Likert scale 1–5) and another on the future intentions (Likert scale 0–4). Higher scores correspond to greater regret and greater intentions to implement concrete pro-environmental behaviors in the future in participants’ daily lives. Finally, two questions (Likert scale 1–5; 1 = Not at all to 5 = very much) were asked to obtain information about the effectiveness and satisfaction perceived by the participants and investigate whether there are differences between the two groups. The Green Behaviors Questionnaire was administered again both at post-test and at follow-up.

### 2.4. Data Analysis

The Statistical Package for the Social Sciences (SPSS, version 30) was used for data processing and analysis (IBM Corp., 2017). Preliminary independent sample T-tests were performed to assess the comparability of the two groups in terms of socio-demographic variables and baseline pro-environmental behaviors. Repeated-measures analysis of covariances (ANCOVAs) were performed using groups as the between variable and time as the within-subject variable. The social desirability scale score was used as a covariate of no interest in the analyses.

Independent sample *t*-tests were conducted (i) to test the difference between the averages of the two groups at the perceived regret scales measured in the post-test; (ii) to test the difference between the averages of the two groups in the perceived liking and effectiveness of the experiment. It also carried out regression analysis between post-experiment psychological outcomes (separately as predictors) and pro-environmental behaviors measured at follow-up (dependent variables).

To reduce the likelihood that the results were influenced by potential confounding variables, additional analyses were conducted. Specifically, to exclude the effect of urbanicity on mobility dimension, we conducted a repeated measures (three time points) ANCOVA between the groups and participant’s residence (urban: >15,000 inhabitants vs. rural: <15,000 inhabitants); moreover, to exclude that results in the purchasing conditions were affected by SES, we performed the analysis by including three related SES subgroups (low, medium, and high). Participants with low SES have a score < 20; participants with medium SES have scores between 20 and 40; participants with high SES have scores between 40 and 66. The classification was based on previous studies that highlighted the impact of SES on pro-environmental behavior (Kollmuss & Agyeman, 2002). Statistical significance for analyses was set at *p* < 0.05.

## 3. Results

The two groups were not statistically different at baseline in terms of socio-demographic variables and pro-environmental behaviors (Table 3).

### 3.1. Repeated Measures ANCOVA

**Figure 2:**
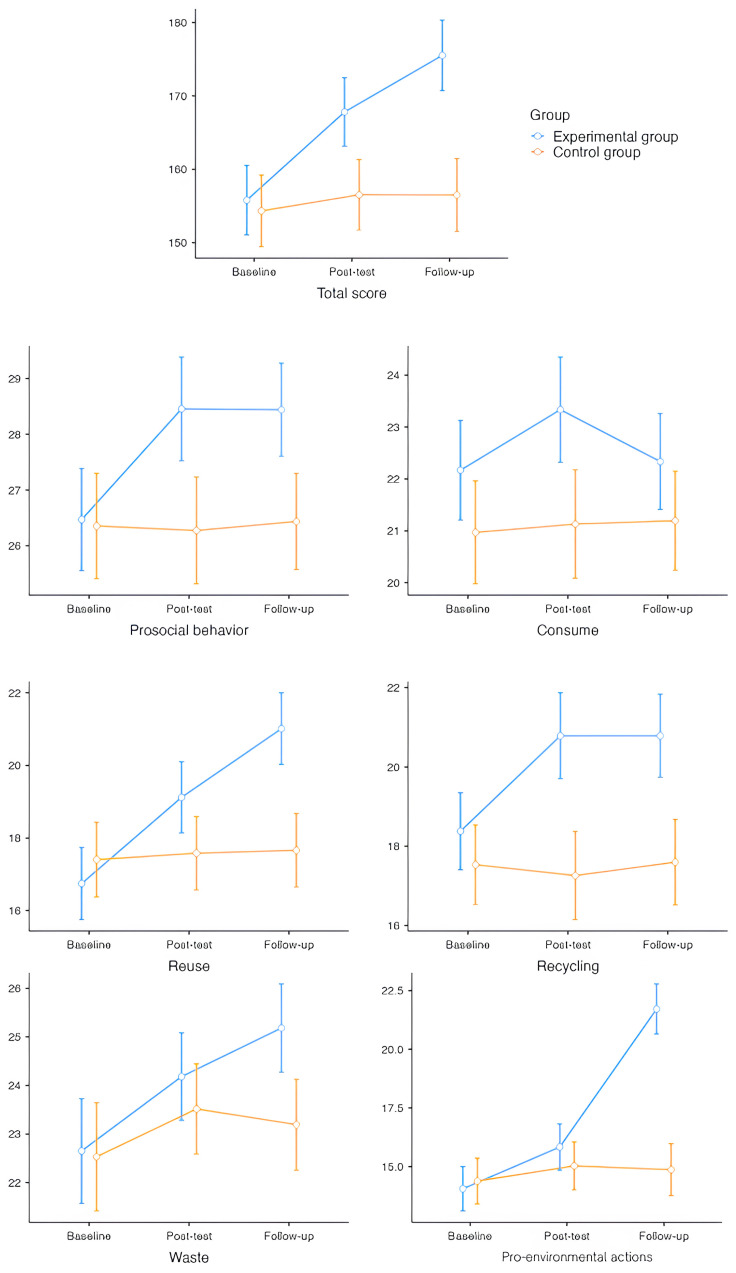
Interaction between the averages of the two groups within the three measurement times of pro-environmental behavior.

The repeated measures ANCOVA, with group as the between-subject factor, time as the within-subject factor, and social desirability as a covariate of no interest, yielded the results shown in Figure 2. We did not include the ANCOVA plots for the purchase and mobility subscales in Figure 2 because no significant changes were observed for these measures.

Regarding prosocial behavior, a main effect of group was observed, F_(1,126)_ = 6.33, *p* = 0.013, with the EG outperforming the CG t_(126)_ = 2.52, *p* = 0.01. No significant main effect of time was observed. There was a significant interaction between group and time, F_(2,252)_ = 7.82, *p* < 0.001, indicating that changes over time differed between the groups. In the CG, no significant changes were observed between time points. However, in the EG, significant improvements were observed. Between baseline and post-test, scores improved significantly, t_(127)_ = −3.389, *p* = 0.002, and further improvements were noted between baseline and follow-up, t_(127)_ = −4.33, *p* < 0.001. At post-test, the EG exhibited significantly higher scores than the CG, t_(126)_ = 3.318, *p* = 0.022. At follow-up, the EG also showed significantly higher scores than the CG, t_(126)_ = 3.24, *p* = 0.018.

No main effects or interaction were observed for purchase in the standard group analysis. However, an explorative analysis including SES as a factor revealed a main effect of SES, F_(2,122)_ = 3.66, *p* = 0.033. Participants from the low SES group showed higher scores than those from the medium SES group.

For consume, a main effect of group was observed, F_(1,126)_ = 5.39, *p* = 0.022, with the EG outperforming the CG, t_(126)_ = 2.32, *p* = 0.022. No significant main effect of time was observed. There was a significant interaction between group and time, F_(2,252)_ = 3.27, *p* = 0.039, so that in the CG there were no significant changes across time, while in the EG, the data showed a trend towards improvement between baseline and post-test, t_(127)_ = 2.68, *p* = 0.085, and a significant decrease between post-test and follow-up, t_(127)_ = 5.67, *p* < 0.01. At post-test, the EG exhibited higher scores than the CG, t_(126)_ = 2.97, *p* = 0.040.

Regarding the reuse subscale, a main effect of group was observed, F_(1,126)_ = 5.91, *p* = 0.016, with the EG outperforming the CG, t_(126)_ = 2.43, *p* = 0.016. A significant main effect of time was also found, F_(2,252)_ = 3.68, *p* = 0.029, with significant improvements between baseline and post-test, t_(127)_ = −3.81, *p* < 0.001, between baseline and follow-up, t_(127)_ = −6.00, *p* < 0.001, and between post-test and follow-up, t_(127)_ = −2.39, *p* = 0.048. The interaction between group and time was significant, F_(2,252)_ = 11.78, *p* < 0.001. In the EG, scores improved significantly between baseline and post-test, t_(127)_ = −5.086, *p* < 0.001, and further between baseline and follow-up, t_(127)_ = −8.16, *p* < 0.001, and between post-test and follow-up, t_(127)_ = −3.34, *p* = 0.014. At follow-up, the EG exhibited significantly higher scores than the CG, t_(126)_ = 4.69, *p* < 0.001.

For recycling, a main effect of group was observed, F_(1,126)_ = 13.90, *p* < 0.001, with the EG outperforming the CG, t_(126)_ = 3.73, *p* < 0.001. No significant main effect of time was observed. There was a significant interaction between group and time, F_(2,252)_ = 11.78, *p* < 0.001. In the CG, no significant changes were observed between time points, while in the EG, scores improved significantly between baseline and post-test, t_(127)_ = −4.455, *p* < 0.001, and further between baseline and follow-up, t_(127)_ = −5.22, *p* < 0.001. At post-test, the EG exhibited significantly higher scores than the CG, t_(126)_ = 4.44, *p* < 0.001, and again at follow-up, t_(126)_ = 4.14, *p* < 0.001.

The 2 × 3 repeated measures ANCOVA for the waste subscale revealed a significant interaction between group and time, F_(2,252)_ = 11.78, *p* < 0.001. No significant main effect of group or time was observed. In the EG, scores improved significantly between baseline and follow-up, t_(127)_ = −5.21, *p* < 0.001, and further between post-test and follow-up, t_(127)_ = −5.65, *p* < 0.001. At follow-up, the EG exhibited higher scores than the CG, t_(126)_ = 3.064, *p* = 0.031.

No main effects or interactions were observed in mobility. However, a significant three-way interaction between group, time, and residence (rural vs. city) was found, F_(2,123)_ = 5.92, *p* = 0.003. The effect of the intervention varied by location, with rural participants in the EG showing a significant increase in scores from baseline to post-test, t_(123)_ = −2.49, *p* = 0.044, and a trend at follow-up suggesting more complex interactions.

Regarding the pro-environmental actions, a main effect of group was observed, F_(1,126)_ = 14.54, *p* < 0.001, with the EG outperforming the CG, t_(126)_ = 3.81, *p* < 0.001. Significant main effects of time were observed, F_(2,252)_ = 5.65, *p* = 0.029. Significant improvements were observed between baseline and post-test, t_(127)_ = −3.74, *p* < 0.001, between baseline and follow-up, t_(127)_ = −11.57, *p* < 0.001, and between post-test and follow-up, t_(127)_ = −10.70, *p* < 0.001. There was a significant interaction between group and time, F_(2,252)_ = 73.73, *p* < 0.001. In the EG, scores improved significantly between baseline and post-test, t_(127)_ = −3.930, *p* = 0.002, and further between baseline and follow-up, t_(127)_ = −15.59, *p* < 0.001, and between post-test and follow-up, t_(127)_ = −3.34, *p* = 0.014. At follow-up, the EG exhibited significantly higher scores than the CG, t_(126)_ = −15.77, *p* < 0.001. For the total score, a main effect of group was observed, F_(1,126)_ = 12.1, *p* < 0.001, indicating differences between the CG and the EG, t_(126)_ = −3.48, *p* < 0.001. The main effect of time was not significant. A significant interaction between group and time was found, F_(2,252)_ = 19.45, *p* < 0.001. In the EG, significant changes were observed between baseline and post-test t_(127)_ = −5.37, *p* < 0.001, baseline and follow-up, t_(127)_ = −8.08, *p* < 0.001, and post-test to follow-up, t_(127)_ = −11.29, *p* < 0.001. In the CG, no significant changes were observed over time. At post-test, the EG exhibited significantly higher scores than the CG, t_(126)_ = 3.31, *p* = 0.002. At follow-up, the EG showed significantly higher scores than the CG, t_(126)_ = 5.42, *p* < 0.001.

### 3.2. Differences Between Groups on Post-Intervention Psychological Outcomes

The EG showed higher scores on the perceived regret scale (t_(126)_ = 3512; *p* < 0.005) than the CG. As shown in Table 4, perceived regret scores predicted several pro-environmental outcomes at follow-up, including total score (β = 0.646; t = 3.074; *p* = 0.02), prosocial behavior (β = 0.098; t = 2.854; *p* = 0.05), reuse (β = 0.126; t = 2.97; *p* = 0.04), and pro-environmental actions (β = 0.16; t = 2.961; *p* = 0.04). In addition, at post-test, the EG reported significantly higher future intention scores than the CG (*p* < 0.01), indicating a stronger willingness to adopt pro-environmental behaviors in the future following the intervention. However, exploratory regression analyses weighted by group did not reveal significant associations between future intentions and follow-up behavioral measures. Finally, the two groups showed no differences in perceived effectiveness and satisfaction.

## 4. Discussion

The present study aimed to examine the effect of anticipated regret on pro-environmental behaviors using regret-based videos in a sample of over 100 young Italian adults. The results of our study provide evidence supporting the role of anticipated regret in motivating pro-environmental behaviors, aligning with previous research (Carfora et al., 2019; Onwezen et al., 2014). This clearly emerges from the group by time interaction on the total score of the Green Behaviors Questionnaire, in which the EG, but not the CG, exhibited higher scores at both post-test and follow-up compared to baseline. Below, we discuss our findings from a short- and long-term perspective, including non-significant effects, observed across all the questionnaire scales.

### 4.1. Short-Term Effects: Baseline vs. Post-Test

In the short term, there was a significant increase in pro-environmental behaviors within the EG between baseline and post-test. In contrast, the CG did not show significant changes. At post-test, the EG scored significantly higher than the CG, supporting the role of regret in promoting behavioral change (Zeelenberg, 1999).

The EG also exhibited a substantial rise in prosocial behaviors, while the CG remained unchanged. This aligns with previous studies suggesting that moral emotions like regret can drive cooperative and prosocial actions (Bandura, 1986) and their interdependence with pro-environmental behavior (Tapia-Fonllem et al., 2013).

Pro-environmental actions within the EG also increased significantly between baseline and post-test. However, no significant differences were observed between the EG and the CG in the short term. Differences emerged only in the long term, suggesting that, while regret can motivate pro-environmental actions, it may take time for such behaviors to manifest as collective action (Van Zomeren et al., 2012). Participants in the EG may have had fewer immediate opportunities to engage in pro-environmental actions, with more pronounced behaviors developing over time.

Recycling behaviors showed marked improvements between baseline and post-test in the EG, while no significant changes occurred in the CG. The EG’s post-test score, significantly higher than that of the CG, indicates that anticipated regret was particularly effective in encouraging recycling. This supports Diekmann and Preisendörfer’s low-cost hypothesis, which suggests that low-cost behaviors, like recycling, are more easily influenced by emotional triggers (Diekmann & Preisendörfer, 1998).

Reuse behaviors also improved within the EG, although no significant differences were found between the EG and the CG at post-test. This may be due to the accessibility and ease of reuse potentially diminishing the intervention’s impact (e.g., peer-to-peer online marketplace or global e-commerce focused on secondhand fashion and accessories). Platforms that reward users economically or socially might encourage continuous behavior change, particularly for younger adults who may see financial and social gains from such platforms (Bocken et al., 2016).

Consumption behaviors, however, did not show significant improvements within the EG between baseline and post-test, although the EG did score higher than the CG at post-test. Consumption choices, particularly for students, are often influenced by deeper values and economic factors (Sheth et al., 2011), making them harder to shift through emotional interventions alone. Additionally, the high baseline score for consumption likely contributed to a ceiling effect, limiting the potential for significant short-term changes.

### 4.2. Long-Term Effects: Baseline vs. Follow-Up

In the long term, the EG exhibited sustained improvements in many pro-environmental behaviors. The total pro-environmental score continued to rise from baseline to follow-up, which supports the theory of regret heuristics that suggests that individuals use past regrets to guide future decisions, leading to lasting behavior changes (Slovic et al., 2002). The CG did not exhibit such changes, highlighting the enduring influence of regret-based interventions.

Prosocial behavior also improved significantly in the EG between baseline and follow-up, further supporting the idea that regret can drive lasting moral motivation (Zeelenberg & Pieters, 2007). Reuse and recycling behaviors followed a similar pattern, with sustained improvements observed in the EG, supporting Diekmann and Preisendörfer’s low-cost hypothesis (Diekmann & Preisendörfer, 1998).

Pro-environmental actions also exhibited continued improvement from baseline to follow-up in the EG. This suggests that the emotional engagement triggered by anticipated regret led to ongoing participation in collective environmental actions (Van Zomeren et al., 2012). Over time, participants likely found more opportunities to engage in pro-environmental actions, such as discussing environmental issues or accessing environmentally relevant content.

Interestingly, waste reduction behaviors only showed significant improvements at the long-term follow-up, suggesting that more complex behaviors may require more time to develop.

### 4.3. Post-Test vs. Follow-Up

Regression analyses indicated that perceived regret at post-test predicted further improvements at follow-up, particularly in pro-environmental behavior, prosocial behavior, reuse, and pro-environmental actions.

The results showed that recycling behaviors remained stable between post-test and follow-up, while reuse behaviors continued to increase. Recycling is often a low-effort, routine action that participants can easily incorporate into their daily lives, as supported by Diekmann and Preisendörfer’s low-cost hypothesis, which suggests that simple, habitual actions like recycling are quickly adopted and require less reinforcement (Diekmann & Preisendörfer, 1998). In contrast, reuse behaviors demand more active decision-making and involve greater personal incentives, such as saving money or earning income from platforms. According to Ajzen’s Theory of Planned Behavior, the complexity and cognitive engagement required for reuse may explain why this behavior continued to grow, as participants gradually integrated it into their habits (Ajzen, 1991). Additionally, the economic and psychological benefits linked to reuse could have reinforced these behaviors more consistently than recycling, which might have already reached a habitual plateau (Lally et al., 2010). Waste reduction behaviors—such as turning off lights, conserving water, and reducing paper use—improved significantly between baseline and follow-up, despite showing no immediate change at post-test. The delayed improvement can also be explained by affective heuristics theory (Slovic et al., 2002), suggesting that, over time, participants developed positive emotional associations with these actions, reinforcing long-term behavior change. In contrast, consumption behaviors, such as using insecticides, air fresheners, and reading product labels, showed initial improvement but declined between post-test and follow-up. This trend can be explained by the seasonality effect, as the post-test occurred in summer when insecticide use is higher, but by autumn (follow-up), the demand for these products had decreased. Additionally, behaviors like reading labels and selecting eco-friendly products require higher cognitive effort, making them harder to sustain without continued reinforcement. According to Ajzen’s Theory of Planned Behavior, these actions demand more deliberate control, and without constant motivation, the intention to maintain these behaviors diminished (Ajzen, 1991).

### 4.4. Non-Significant Results: Purchase and Mobility

No significant changes were found in purchasing behaviors in either group, although SES played a role. Participants with lower SES reported greener purchasing behaviors, likely due to necessity rather than environmental concern (Stephens, 2007). Financial barriers faced by middle-class participants may have further hindered their ability to adopt green practices. In this dimension, an inconsistency with Carfora et al. was observed, which may be explained by differences in the conceptualization and measurement of pro-environmental behaviors (Carfora et al., 2019). A key distinction in our study is the separation of “purchase” behaviors from “consume” behaviors, allowing for a clearer understanding of how different actions contribute to environmental outcomes. For example, the environmental impact of purchasing red meat occurs at the point of purchase, regardless of whether the individual consumes it. This impact persists even if the product is discarded and can be further amplified if it is wasted. Conversely, consumption behaviors, such as using chemical air fresheners or pesticides, generate environmental impacts at the point of use, independent of the purchase. Moreover, Carfora et al. did not account for the influence of autonomy over purchasing decisions, which could be an important factor in shaping pro-environmental behaviors (Carfora et al., 2019). Our sample was predominantly composed of students living with their parents and not frequently engaged in purchasing. In contrast, the study by Carfora et al. did not provide specific details on the living arrangements of their participants, but it is likely that the Milan sample had a higher proportion of students living away from home (Carfora et al., 2019). This difference in living arrangements could be significant because students living independently may have more autonomy over their purchasing decisions, making anticipated regret a more potent motivator for behavior change. Mobility behaviors also did not show significant improvements, consistent with the high-cost hypothesis (Diekmann & Preisendörfer, 1998). Changes in mobility often involve substantial lifestyle adjustments, such as switching from car use to public transportation. It would be interesting to further disambiguate the exploratory analysis, which revealed that rural participants (a minority compared to urban residents) in the EG showed greater improvements, in line with Berenguer et al., who found that those living in cities hold more environmental responsibility values but show a less pro-environmental orientation when attitude and behavioral intentions scales are used (Berenguer et al., 2005). People living in rural contexts present greater attitudes of environmental responsibility and greater coherence in expressing behavioral intentions compatible with environmental protection also in terms of travel.

## 5. Limitations and Future Implications

Our study had several limitations. First, recruitment was conducted through a convenience sample, consisting exclusively of university students recruited from a single Italian university. This may limit the generalizability of the findings to other populations. Future studies should replicate the intervention with more diverse samples, including older adults and adolescents from different sociocultural backgrounds. Second, pro-environmental behaviors were measured through self-report questionnaires. Although statistically controlled, self-report measures could still be affected by response bias or discrepancies between intentions and actual behavior. In the future, they could integrate behavioral or observational measures, such as actual purchasing decisions, energy consumption monitoring, or mobility tracking (Lange & Dewitte, 2019). Third, the intervention period was relatively short (ten days). Although a three-month follow-up was conducted, longer follow-ups would be necessary to determine whether regret-based interventions produce stable long-term behavioral change. This limitation was aligned with Świątkowski et al. (2024): although behavioral interventions can promote pro-environmental actions, their long-term stability remains insufficiently explored across contexts and populations. Finally, some behavioral domains, particularly mobility and purchase, appeared resistant to change. These behaviors are often constrained by economic resources and infrastructure, suggesting that emotional interventions alone may not be sufficient to influence more costly environmental behaviors.

Despite the aforementioned limits, the present findings contribute to the growing literature on the psychological determinants of pro-environmental behavior by providing empirical support for the role of anticipated regret as a motivational mechanism. The study underscores the role of affective heuristics, as proposed by Slovic and colleagues, in fostering long-term reflection and behavioral shifts (Slovic et al., 2002). In line with that, the results suggest that emotions can facilitate behavioral change by making the future consequences of environmentally harmful actions more psychologically salient. Moreover, the study extends previous research on anticipated regret by demonstrating that its influence is not limited to specific behaviors (e.g., dietary choices), but can generalize across multiple domains of sustainable behavior, including prosocial actions, recycling, reuse, and pro-environmental actions. This supports the idea that regret may function as a cross-domain motivational driver in sustainability-related decision-making (Zeelenberg & Pieters, 2007).

From an applied perspective, our results suggest that digital interventions based on anticipated regret may represent an effective strategy to promote sustainable behaviors. The EkoTok format, consisting of brief emotionally framed videos delivered through automated messaging systems, offers several practical advantages. It is easily disseminated through smartphones, requires minimal resources, and can be integrated into educational programs. Educational institutions and policymakers could adopt similar interventions to complement traditional environmental education programs. This approach could also be adapted to other domains of behavior change beyond environmental sustainability, including health behaviors, responsible consumption, and civic engagement, where emotional anticipation may play a critical role in motivating long-term behavioral commitment.

## 6. Conclusions

The findings of this study demonstrate that anticipated regret can effectively motivate pro-environmental behaviors, particularly low-cost and habitual actions such as recycling, reuse, and prosocial behaviors. Our results support the hypothesis that behaviors requiring minimal psychological or physical effort are more easily influenced by emotional triggers. In contrast, higher-cost behaviors such as mobility and consumption were more resistant to change, likely due to the greater effort or structural adjustments they require. These results highlight the importance of designing interventions that address specific barriers, such as SES and the complexity of the behavior itself, to facilitate sustainable behavior change. The effectiveness of EkoToks can be understood considering the “non-tangibility of consequences” often associated with cognitive limitations that impact environmental choices. To explore the internalization of affective heuristics, future research could use scenarios in which participants imagine how they would act and feel in emotionally charged situations. While this method is efficient and can evoke strong emotions, it has limitations. The decisions made in imagined situations may not reflect real-life choices, and social desirability might also influence responses. Although social desirability was controlled for in this study, future work could benefit from testing implicit responses to pro-environmental behaviors. EkoToks—used for the first time in this study and well-received by both the EG and CG—proved to be effective in increasing pro-environmental behaviors, including prosocial behavior, reuse, recycling, waste reduction and on pro-environmental actions. Therefore, this strategy could be applied at an individual level and easily disseminated via smartphones to promote multiple dimensions of sustainable behavior. Considering this psychological mechanism, it would be valuable to test the use of immersive or mixed virtual reality paradigms for EkoTok delivery, in order to make the consequences of one’s behaviors more “tangible” across all behavioral domains considered.

## Figures and Tables

**Table 1:** Characteristics of sample. SES: socio-economic status; data are N (%) or mean (SD).

Characteristics	CG	EG	Total	*t*-Test	*p*
N	62 (48%)	66 (52%)	128		
Female	46 (74%)	45 (68%)	91 (71%)	0.75	0.457
Mean Age	22 (2.9)	23 (2.9)	23 (2.9)	0.64	0.521
Mean SES	34 (13.8)	35 (13.4)	35 (13.5)	−0.45	0.651
Living in urban areas (>15,000)	49 (79%)	43 (65%)	92 (72%)		
Do you live with your parents? (Yes)	50 (81%)	50 (76%)	100 (78%)		
Do you take care of the shopping yourself? (Not Frequently)	40 (65%)	38 (58%)	78 (61%)		

**Table 2:** Green Behaviors Questionnaire. Items marked with an asterisk are reverse scored.

If possible, I give something to beggars. In a self-service restaurant, I leave the tray on the table. * If there is an elderly or disabled person on a bus, I give up my seat. If a close relative stayed in the hospital for a week or two for minor surgery, I would go to visit them. When I leave a place, I greet the people I meet. If someone with me needs help, I gladly help them. I feel uncomfortable having neighbors from other countries. * I do not use public transport without paying for a ticket. I buy local fruit and vegetables. I buy seasonal fruit and vegetables. I buy locally caught fish I buy fish that are protected or subject to fishing restrictions. * I buy free-range eggs. I buy red meat more than twice a week. * I buy organically grown vegetables. I buy foods with the closest expiration date first. If there are insects in the house, I kill them with a chemical insecticide. * I use chemical air fresheners (e.g., in the bathroom). * I read product labels carefully before using them. I use rechargeable batteries. I prefer paper bags to plastic ones. When I go shopping, I bring my own bag. When I go shopping, I only buy what I need. I plan my shopping before going to the supermarket. When an object breaks, I try to repair it. When an object breaks, I throw it away. * When an object breaks, I buy a new one. * I reuse water bottles. Sometimes I sell goods that I no longer use. Sometimes I give away goods that I no longer use. Sometimes I borrow goods that I use occasionally instead of buying them. Sometimes I lend goods that I use occasionally. I buy products with minimal packaging (e.g., products packaged with very little plastic). I usually use disposable products. * I usually buy water in returnable bottles. I reuse empty product containers. I separate waste for recycling. I return unused medicines to the pharmacy. I am not careful to separate the different parts of the packaging before throwing them away. * I pay attention to storing food properly so that it can be consumed later. I do not leave the heating or air conditioning on when I am not at home. I do not leave the lights on when I move from room to room. I turn off the tap while brushing my teeth. Before taking a shower, I let the water run for a long time to ensure it reaches the right temperature. * I shower more often than I take baths. I turn off my computer when it is not in use. I use the printer only when necessary. When writing, I use both sides of the paper. I travel by bike. I travel by car. * I travel by public transport. I get around on foot. I get around by scooter. I do not use the car for short trips. I do not use the car in the city. For long journeys, I only take the plane even when alternative options (e.g., trains) are available. * I sometimes argue with my friends about environmental issues. I am a member of an environmental association. I have argued with someone about their environmentally harmful behavior. I sometimes throw rubbish on the ground. * I sometimes throw rubbish into the sea. * I am interested in environmental issues (e.g., reading articles, watching videos). I donate money to environmental associations. I vote for political candidates who support environmental issues.

**Table 3:** Descriptive statistics of measures between groups at three time points. ns = Not significant.

Time	Measures	CG	EG	*p*-Value
Baseline				
	Prosocial Behavior	26.35 (3.23)	26.47 (4.18)	ns
	Purchase	20.97 (4.67)	21.15 (4.34)	ns
	Consume	20.97 (4.13)	22.17 (3.74)	ns
	Reuse	17.4 (4.08)	16.74 (4.085)	ns
	Recycling	17.53 (4.32)	18.38 (3.65)	ns
	Waste Reduction	22.53 (4.62)	22.65 (4.21)	ns
	Mobility	14.18 (4.42)	14.17 (3.96)	ns
	Pro-environmental actions	14.39 (4.11)	14.06 (3.58)	ns
	Total score	154.32 (20.07)	155.79 (18.83)	ns
	Social Desirability	17.26 (5.07)	17.97 (5.16)	ns
Post-test				
	Prosocial Behavior	26.27 (4.22)	28.45 (3.37)	*p* < 0.005
	Purchase	20.89 (4.342)	21.5 (3.919)	ns
	Consume	21.13 (4.604)	23.33 (3.672)	*p* < 0.01
	Reuse	17.58 (3.766)	19.12 (4.241)	*p* < 0.05
	Recycling	17.26 (4.289)	20.79 (4.566)	*p* < 0.001
	Waste Reduction	23.52 (3.444)	24.18 (3.898)	ns
	Mobility	14.5 (4.032)	14.59 (3.464)	ns
	Pro-environmental actions	15.03 (4.49)	15.83 (3.506)	*p* < 0.005
	Total score	156.52 (19.498)	167.8 (18,743)	*p* < 0.001
	Perceived Regret	29.55 (9.77)	33.42 (7.66)	*p* < 0.001
	Future Intention	45 (8.2)	47 (8.3)	*p* < 0.01
	Effectiveness	2.97 (0.84)	2.97 (0.89)	ns
	Satisfaction	2.90 (0.78)	3.05 (0.86)	ns
Follow-up				
	Prosocial Behavior	26.32 (3.41)	28.43 (3.37)	*p* < 0.001
	Purchase	20.92 (3.97)	21.46 (3.96)	ns
	Consume	21.01(3.97)	22.32 (3.69)	ns
	Reuse	17.49 (3.94)	21 (4.4)	*p* < 0.001
	Recycling	17.39 (3.94)	20.78 (4.58)	*p* < 0.001
	Waste Reduction	23.02 (3.53)	25.17 (3.91)	*p* < 0.005
	Mobility	14.33 (4.02)	14.60 (3.44)	ns
	Pro-environmental actions	14.71 (3.88)	21.70 (4.77)	*p* < 0.001
	Total score	156.48 (19.47)	175.5 (19.96)	*p* < 0.001

**Table 4:** Regression analyses predicting follow-up pro-environmental behaviors. ns = not significant.

Measures	Β	SD	Standardized Regression Coefficient	*t*	*p*-Value (Uncorrected)	*p*-Value (Corrected)
Prosocial Behavior	0.098	0.034	0.246	2.854	0.005	0.045
Purchase	−0.001	0.04	−0.002	−0.026	0.979	ns
Consume	0.071	0.038	0.167	1.897	0.06	ns
Reuse	0.126	0.042	0.256	2.97	0.004	0.036
Recycling	0.065	0.045	0.127	1.441	0.152	ns
Waste Reduction	0.085	0.038	0.195	2.233	0.027	ns
Mobility	0.018	0.037	0.043	0.486	0.628	ns
Pro-environmental actions	0.16	0.054	0.255	2.961	0.004	0.036
Total score	0.646	0.21	0.264	3.074	0.003	0.024

## Data Availability

The datasets used and analyzed during the current study are available from the corresponding author on reasonable request.

## Abbreviations

The following abbreviations are used in this manuscript:

ANCOVA(s)	Analysis of covariance(s)
CG	Control group
EAT-26	Eating Attitudes Test-26
EG	Experimental group
ES	Effect size
MC-SDS	Marlowe–Crowne Social Desirability Scale
N	Sample size
SAS	Self-Rating Anxiety Scale
SD	Standard deviation
SDGs	Sustainable Development Goals
SES	Socio-economic status
SPSS	The Statistical Package for the Social Sciences
A	Cronbach’s alpha
Ω	McDonald’s omega

## Appendix A

**Table A1:** EkoTok phrases said only to the EG are shown in **bold**.

N	Measures	Phrases
1	All Dimensions	All groups: “The concentration of CO_2_ causes a global rise in temperature which in turn makes floods, droughts, hydrogeological instability, the spread of diseases, crises in agricultural systems, water crises and the extinction of animal and plant species more and more frequent. Consumption choices and daily behaviors (such as recycling, reuse, mobility and waste reduction) have a constant impact on the environment”. **EG: You may regret not reducing your CO_2_ emissions**, for example by carefully choosing the products to purchase and consume, recycling using products rather than throwing them away, reducing waste of domestic energy or car use in the city especially for short trips. Think about all this. See you tomorrow!
2	Waste	All Groups: energy use is responsible for 77.1% of greenhouse gas emissions and therefore global warming; domestic uses account for 29% of world energy consumption and therefore global warming, in particular technological equipment. **EG: You may regret not reducing domestic water or energy consumption** (for example by leaving lights on in unusable rooms or not turning off the PC if not in use); in fact, higher temperatures increase mortality, risk of severe droughts and forest fires, sea level rise and ocean acidification. See you tomorrow!
3	Mobility	All groups: At this moment there are millions and millions of trains, ships, planes, cars, trucks, buses, trams, motorbikes… We all move, constantly. According to a recent estimate, in 2019 in Italy alone, road transport contributed 23% to greenhouse gas emissions, of which almost 70% was attributable to cars… There are contexts in which, as we know, using the car is essential, but it is worth trying to reorganize those areas of your daily life where, with different measures, you can do without them. In fact, fuels, in addition to weighing on your wallet, contribute heavily to air pollution. **EG: You might feel regret in not reducing these emissions in favor of greater sustainable mobility**, for example by favoring public transport, pedestrian and cycle mobility, electric vehicles or shared mobility through car sharing apps (rental of short-term vehicles) or carpooling (sharing a private car with other travelers in order to reduce travel costs) Have a good trip… See you tomorrow!
4	Consumption	All groups: Not only human beings but also all the objects created by them, bought, and used involve the consumption of energy and resources and cause carbon emissions during all phases of the production, transport, sale, use and disposal cycle. Sustainable products have a low ecological footprint and are characterized by: …aspects are guaranteed by the presence of sustainability certifications of the products on the labels. **EG: You might feel regret at not consuming sustainable products by not reading the label carefully before using them or even before purchasing them,** to verify that all the information necessary to make ethical and ecologically responsible consumption choices is present. See you tomorrow!
5	Purchasing	All groups: Buying seasonal products has multiple benefits for consumers and the planet. Knowing the value of a product is not just about the price, but the origin and characteristics are of vital importance for responsible consumption. EG: **You may have great regrets in not purchasing seasonal products or in not carefully reading the characteristics of the product you purchase.** See you tomorrow!
6	Recycling, Consumption, Reuse	All groups: More than half of the tons of rubbish produced ends up in landfill, and the less degradable the components of each product are, the slower they will be to decompose, the longer they will remain in landfill sites, emitting greenhouse gases (90–98% methane and carbon dioxide) and, unfortunately, favoring climate change one of the most powerful weapons against pollution, an ally of environmental sustainability is you… **EG…you may have regrets** **in not reducing your waste production** (e.g., buying only what you need at the supermarket and planning your shopping list in advance) in not reusing your possessions instead of throwing them away or for example by taking advantage of recent apps to resell things you no longer use in not always and correctly recycling waste. Separate waste collection guarantees, in addition to a more efficient use of resources, also benefits the environment and the economy. The savings achieved are electricity, water and raw materials. See you tomorrow!
7	Purchasing	All groups: From the 1950s to today, meat consumption per capita has doubled. Half a billion animals are killed every day All of this is having major environmental repercussions. **EG: You may have regrets about often purchasing red and processed meat, given that:** (1) sheep and cattle produce methane gas, which strongly contributes to global warming. (2) intensive farming contributes to deforestation. (3) the classic Western meat-based diet requires the use of almost 16,000 L of water per day, while a plant-based diet requires only 1100 L. All this negatively affects global warming and will lead to large periods of drought See you tomorrow!
8	Waste, Reuse	All groups: More than a third of the food produced worldwide is thrown away. The waste is calculated at 1.4 billion tons of food products and 800 billion dollars. In addition to the waste of still edible foods, the resources to produce them are also wasted (energy, water, land use, fuel, workforce). On an economic level, approximately one trillion dollars of social, health and environmental costs caused by food waste must be accounted for. Loss of valuable biodiversity. **EG: You might feel great regret at wasting and or throwing away food** rather than reusing it or by organizing a responsible and well-organized purchase beforehand or by consuming the foods with the closest expiration dates first… See you tomorrow!
9	Pro-environmental actions	All groups: Every year it is estimated that unfortunately 4 to 12 million tons of plastic end up in the seas around the world, causing 80% of marine pollution. Bottles, bags, packaging, fishing nets, cigarette butts, pesticides… most of the waste produced by man ends up in one way or another in the sea, causing serious consequences for the health of waters, marine animal life and plants, producing perhaps irreparable damage to the food chain. **EG: You may have great regrets about throwing waste into the sea or about not stopping someone else from doing so** It’s not just a matter of mess and dirt. Plastic waste injures animals who may become trapped in the larger pieces or may even mistake the smaller parts for food. Ingesting plastic particles prevents the digestion of normal foods and can promote the presence of toxic chemical pollutants in their body; furthermore, humans eat plastic ingested by fish through the food chain. The effects this step has on human health are still unknown. Plastic waste also causes economic loss to those sectors and communities that depend on marine products. See you tomorrow!
10	All dimensions	“The concentration of CO_2_ causes a global rise in temperature which in turn makes floods, droughts, hydrogeological instability, the spread of diseases, crises in agricultural systems, water crises and the extinction of animal and plant species more and more frequent. Consumption choices and daily behaviors (such as recycling, reuse, mobility and waste reduction) have a constant impact on the environment”. Thank you for your participation; remember the importance of what you saw. **EG: All I must do is make one last choice: See you tomorrow! Or see you tomorrow?** **You might regret your choice.**
